# Residual Stress of a TC17 Titanium Alloy after Belt Grinding and Its Impact on the Fatigue Life

**DOI:** 10.3390/ma11112218

**Published:** 2018-11-08

**Authors:** Yi He, Guijian Xiao, Wei Li, Yun Huang

**Affiliations:** The State Key Laboratory of Mechanical Transmissions, Chongqing University, Chongqing 400044, China; m18423397236@163.com (Y.H.); 13452455686@163.com (W.L.); yunhuang@samhida.com (Y.H.)

**Keywords:** TC17 titanium alloy, belt grinding, residual stress, fatigue failure

## Abstract

Titanium alloy materials are widely used in the design of key parts, such as aeroengine blades and integral blades. The surface residual stress has a great influence on the fatigue life of the parts mentioned above. Presently, abrasive belt grinding can form residual stress on the surface. However, the formation mechanism has not yet been revealed, providing the impetus for the present study. First of all, the surface residual stress is characterized based on Bragg’s law. The influence of contact force, reciprocating frequency, and feed speed on the residual stress of a titanium alloy abrasive belt grinding is obtained using an experimental method. The residual stress model is simulated by the tensile force on the surface of the model, and the fatigue life of the bar under a sinusoidal tensile load is analyzed by simulating the fatigue test of the titanium alloy bar. Finally, fatigue testing and fracture analysis are carried out. The experimental results show that with the increase of the grinding contact force, increase of the reciprocating frequency, and decrease of the feed speed, the residual compressive stress on the surface of the parts increases and the fatigue life is higher at the same working stress level. It also shows that the residual compressive stress produced by abrasive belt grinding is in the range of 120–300 MPa. The fatigue simulation curve’s inflection point appears at the level of 550 MPa. The error between the simulation data and the experimental data is less than 10%, which shows the accuracy of the simulation experiment. The fracture morphology at room temperature is a ductile fracture with fine equiaxed dimples.

## 1. Introduction

Titanium alloys were developed in the middle of the 20th century and are key metal materials, because titanium has a lower density, better corrosion resistance, and higher strength than many other metals. They are widely used in the design and manufacture of aeroengine compressor blades, integral blades, and fan blades, because of their high heat resistance and other excellent properties [1]. China’s Shenzhou series spacecraft and the recent, independently developed domestic passenger plane C919 indicate that China has become a significantly space-focused country, and the amount of titanium alloys used in the aerospace and other manufacturing fields is increasing year by year, with alloys playing an increasingly important role. It has become an important basis for judging the advanced nature of major aerospace parts and equipment; the service life of these key parts made from titanium alloys is closely related to the service life and quality of these important achievements, and the most common failure form is fatigue failure [2] (low cycle fatigue and high cycle fatigue) under service conditions. Therefore, it is of great practical significance to analyze the fatigue life of titanium alloys.

As is well-known, the reasonable distribution of residual stress on mechanical parts is of great significance to improve its performance. It is especially suitable for the components bearing an alternate load [3]. Bhaumik et al. [4] found that about 60% of the in-service faults of aerospace components are caused by fatigue, and the surface integrity characteristics, such as residual stress, have a great influence on the fatigue life of aeronautic and spaceflight components. It is very important to explore the influence law guiding engineering practice. However, the mechanism of the influence of abrasive belt grinding on the fatigue life of titanium alloys has not been grasped presently. Therefore, it is difficult to guide the formulation of the evaluation standard of surface characteristics for the optimization of the fatigue life of titanium alloys.

In their research on surface integrity in titanium alloy grinding, Bigerelle et al. [5,6] proposed a method to characterize surface roughness in the process of tool wear, and a series of roughness process parameters were used to characterize the surface integrity, processing dynamics, and mechanical properties. In addition, the fractal model of the wearing process in polishing and the model of the machining process with polishing were established. Jourani et al. [7] studied the relationship between the abrasive belt structure and surface quality, and analyzed the physical mechanism of abrasive belt grinding and polishing. The mathematical model of the process of material removal on the surface of the parts was established. Segreto [8] applied the traditional extraction method based on statistics, and the feature extraction method based on wavelet packet transform to the sensor signal detected in the polishing process. The surface roughness level of polished parts was determined. Eriksen et al. [9] combined the traditional tool manufacturing process and a new robot-assisted polishing method to form multifunctional surfaces.

Xiao et al. [10] analyzed the influence of the abrasive belt size and rough grinding pressure on the grinding surface roughness, work hardening, metallographic structure, and residual stress of a titanium alloy blade’s abrasive belt using experiments, and established a regression model of the surface roughness. Zhao [11] studied the prediction of the blade surface roughness and the model of parameter optimization in the polishing of flexible abrasive tools. The model obtained by the experiments could improve the surface roughness by 25%. Xiao et al. [12,13] studied the adaptive belt grinding method for the edge of aeronautical blades, and analyzed the surface roughness after belt grinding. The results showed that after belt grinding, the surface roughness is less than 0.25 μm, and the surface is under compressive stress. Dong et al. [14] ground a titanium alloy by using two kinds of microcrystalline corundum grinding wheels with large porosity and common porosity, respectively. The experimental results show that the large-porosity grinding wheel could obtain a better surface quality.

The research on surface integrity and fatigue life includes fatigue failure types, such as high and low cycle fatigue, bending fatigue, rolling contact fatigue, and so on.

Jeelani and Ramakrishnan [15] studied the machined surface integrity and fatigue life of a Ti-6Al-2Sn-4Zr-2Mo titanium alloy. The results showed that there were many defects on the machined surface. However, the surface integrity quality of titanium alloys can be improved, and thus the service life of the part can be improved. This is due to the formation of residual compressive stress on the surface of the part, which can reach 275 MPa. The surface integrity and fatigue life of a machined Ti-Al alloy were also studied by Mantle et al. [16] The results show that the microcracks (with a depth less than 20 μm) produced in the turning process of the titanium alloy can reduce the life of the parts. In recent years, Lee et al. [17] and Bhaumil et al. [18] calculated the effect of roughness on the contact fatigue using mesoscopic methods. The results showed that the fatigue properties of the materials with a roughness greater than 0.4 μm will be significantly affected. James et al. [19] found that when the average stress is lower than the yield point of 20 MPa, there is a linear relationship between residual stress and the number of experimental cycles to fatigue.

Huang et al. [20] studied the effect of different surface integrity qualities on the low cycle and high cycle fatigue life of a GH33A superalloy at high and normal temperatures, which provided the experimental data for the design of a new engine turbine disk. Sun and Huang [21] studied the effect of different surface working conditions on the fatigue life of a high-strength titanium alloy. The results showed that the fatigue strength of the high-strength titanium alloy increased by about one third when a surface compressive stress layer of 250 μm was introduced. At the same time, the fatigue resistance was increased by about 70% when the surface was smooth and flat without defects. Li Kang et al. [22] modified the surface of a TC4 titanium alloy using wet shot peening. The fatigue crack initiation position of the specimen was transferred from the surface to the region, 1 mm deep inside the specimen, thus the fatigue life of the material was improved significantly. Wang Xin et al. [23] used ceramic shot peening to treat the ground surface of an alloy. The results show that the fatigue limit of alloy grinding decreased from 583 MPa to 465 MPa at 650 °C when the stress concentration factor was increased from Kt = 1 to Kt = 1.7. After high-strength shot peening, the fatigue limit of Kt = 1.7 returned to 530 MPa, and the low-strength shot peening had no effect on the fatigue limit of Kt = 1.7. Zhou [24] applied the research results of a laser impact composite strengthening mechanism to the turbine blade of a certain aeroengine, and focused on the application of turbine blade tenon. The fatigue life of the turbine blade at high and low temperatures was improved by 379%.

From the above, it can be seen that the research on the influence of the surface integrity and of the surface integrity on the fatigue life of titanium alloy abrasive belt grinding has been focused on the surface texture optimization, such as the working environment temperature, surface roughness, and so on. However, there is little research on residual stress on the surface of titanium alloy grinding, so it is difficult to understand the mechanism of the residual stress on the fatigue life of the grinding surface of the titanium alloy abrasive belt. The residual stress of the TC17 titanium alloy after belt grinding and its impact on fatigue life is presented here. In this study, we reveal the influence of the three main grinding parameters on residual stress, establish the characterization model of residual stress, predict the fatigue life of the TC17 bar after grinding by simulation, and carry out a fatigue experiment. The influence of various parameters on fatigue life is studied, and the experimental fracture surface is analyzed and discussed, which provides a reference for the influence of the fatigue life of titanium alloys after grinding with an abrasive belt.

## 2. Materials and Methods

### 2.1. Materials

The chemical composition and physical properties of the TC17 material are shown in Table 1 and Table 2, as follows:

The TC17 alloy is a kind of α/β-type hot strong titanium alloy with excellent comprehensive properties and is rich in the β-phase, which has a high strength, good fracture toughness, large quenching depth, wide stable range of forging, low thermal conductivity, and high cutting temperature. However, it also has some disadvantages, such as a low elastic modulus, easy deformation, high grinding force, serious tool wear, and cold hardening [25,26].

### 2.2. Methods

The surface of a TC17 alloy bar was machined with different abrasive belt grinding process parameters, and the residual stress values of the titanium alloy surface under different technological parameters were measured and were compared with each other. Finally, the conclusion was drawn.

**Figure 1:**
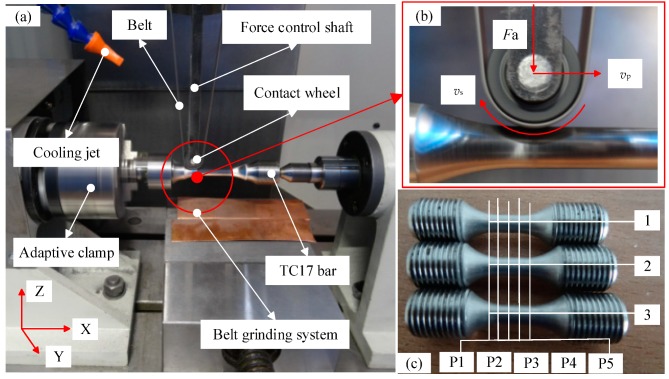
Experimental device and experimental materials: (**a**) grinding device with titanium alloy rods; (**b**) belt grinding system; (**c**) distribution of the five test points.

The experimental device is shown in Figure 1, as follows: Figure 1a is a grinding device with titanium alloy rods, which is composed of a belt (rough grinding with Al_2_O_3_ abrasive grain, 50 μm and finish grinding with SC abrasive grain, 20 μm), force control shaft, cooling jet, contract wheel, adaptive clamp, TC17 bar, and belt grinding system. The two ends of the TC17 bar are rotated by the adaptive clamp to finish the circumferential grinding. At the same time, the axial grinding of the force control shaft is carried out along the axial motion. Figure 1b is the belt grinding system. In Figure 1b, *F*_a_ is the force of the belt on the workpiece, *v*_p_ is the feed speed, and *v*_s_ is the grinding speed. Figure 1c shows the distribution of the five test points where the residual stress of each workpiece was measured. Each group consisted of three workpieces.

## 3. Analysis of Surface Residual Stress Characteristics

### 3.1. Characterization of Surface Residual Stress Based on Titanium Alloy Belt Grinding

**Figure 2:**
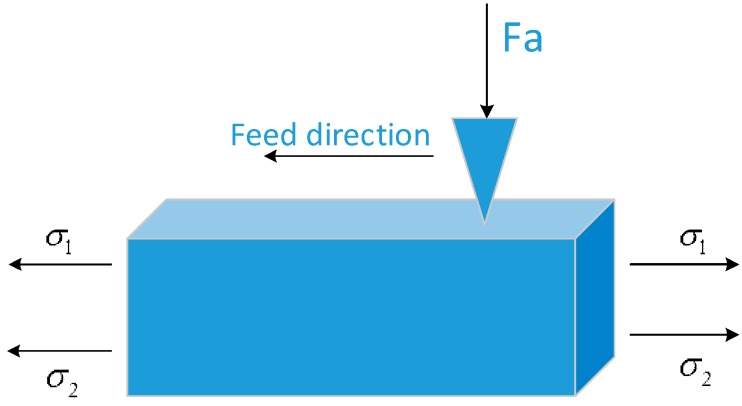
Stress load during single abrasive grinding.

In the grinding process, the abrasive is extruded with the workpiece under the normal grinding pressure, and is fed along the axial direction at the same time, as shown in Figure 2. In this process, because of the grinding heat and the direction of the abrasive feed extrusion, the stresses *σ*_1_ and *σ*_2_ are produced by the grinding heat and extrusion, respectively, and appear as a residual tensile stress state. The residual compressive stress produced when the grinding force *F*_a_ is combined with these two stresses to obtain the final surface residual stress distribution. When the residual compressive stress produced by *F*_a_ is greater than *σ*_1_ and *σ*_2_, the surface presents a state of compressive stress. Otherwise, there will be a residual tensile stress state. This is why the residual stress distribution in different states can be obtained by controlling the grinding pressure in abrasive belt grinding.

For titanium alloy polycrystalline materials, the corresponding macroscopic strain of residual stress is considered to be the sum of the lattice strain. Therefore, residual stress can be calculated by measuring the lattice strain, based on Hooke’s law and the elastic mechanics theory. The magnitude of residual stress can be calculated using the X-ray diffraction method based on Bragg’s law.

(1) n⋅λ=2d⋅sinθ,

where *n* is the integer representing the diffraction order, *λ* is the wavelength of the X-ray, *d* is the crystal plane spacing of the crystal plane, and *θ* is the diffraction angle. When the contact force, *F*, and stresses, *σ*_1_ and *σ*_2_, cause the lattice spacing, *d*, to change, the diffraction angle, *θ*, changes, so the change of *d* can be obtained by measuring *θ*, and the magnitude of stress in a certain direction can be obtained according to the elastic mechanics formula, as follows:

(2) $$\sigma_{x}=K\cdot M,$$

where *K* is the stress constant and *M* is measured experimentally.

(3) $$K=-\frac{E}{2(1+\mu )}\cdot ctg\theta_{0},$$

(4) $$M=\frac{\partial \left(2\theta \right)}{\partial (sin^{2}\psi )}.$$

In the above equations: 

K—Stress constant;

E—Elastic modulus of the materials;

*μ*—Poisson’s ratio of the measured crystal planes of materials;

$\theta_{0}$—Diffraction angles of materials without stress;

M—Slope of 2θ to $sin^{2}\psi$;

*ψ*—Angle between the normal direction of the crystal diffraction plane and the normal line of the sample surface [27].

For the characterization of residual stress, the forms of residual stress are different according to the different service environments of the different components. For example, the residual stress induced by the shot peening of the mechanical parts is affected by many technological parameters, including the type, size, hardness, and velocity of the projectile injection, in addition to being related to the material of the shot-peening part. The distance between the nozzle and spray surface, injection angle, shot-peening time, and coverage rate are mutually restricted. In order to measure the comprehensive effect of these parameters on residual stress, the magnitude of residual stress is usually characterized by the shot-peening strength. In the study of the rolling contact properties of materials, the influence of the gradient, magnitude, and depth of residual stress on the rolling contact properties is usually selected.

**Figure 3:**
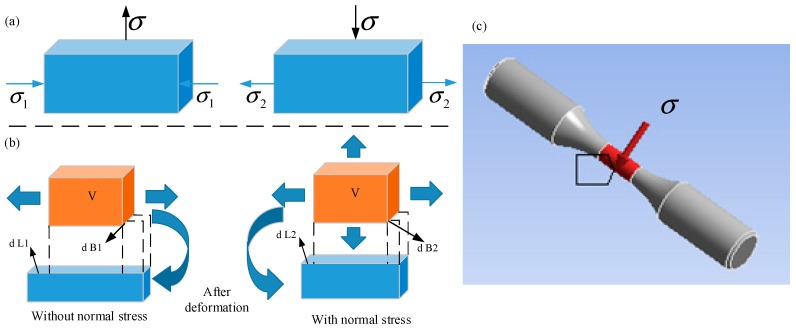
Residual stress simulation: (**a**) stress’s effect on the element; (**b**) deformation of the element; (**c**) model with normal stress on the middle surface.

The finite element method is a very popular method that has been widely used in many fields [28,29,30]; thus, we have used this method in the current paper. In the simulation experiment, normal stress of a different magnitude is applied on the outer surface of the bar to characterize the residual stress of a different magnitude. When the stress is applied on the surface, the stress’s effect on the element is as shown in Figure 3a. The distribution of the residual stress on the surface of the material is obtained by applying the normal tensile stress and the compressive stress to the surface of the material, respectively. The stress and deformation of the hexahedron mesh on the surface of the bar, subjected to tensile force, and the situation after the tensile stress loading can be seen in Figure 3b. We can clearly see that the total deformation of the hexahedron is reduced (dL1 > dL2 and dB1 > dB2) because of the stress of the upper and lower surfaces, the damage of the tensile work is reduced, and the number of fatigue cycles is increased. So, the residual compressive stress is simulated using tensile stress in this simulation experiment. Figure 3c shows the model with normal stress on the middle surface in the ANSYS Workbench software (ANSYS Workench 14.5, ANSYS, Canonsburg, PA, USA).

### 3.2. Influence of Grinding Parameters on Surface Residual Stress

The Panalytical X-ray projector was used to measure the surface residual stress after belt polishing, and the repetitive positioning accuracy of the goniometer and minimum step was 0.0001 degrees.

**Figure 4:**
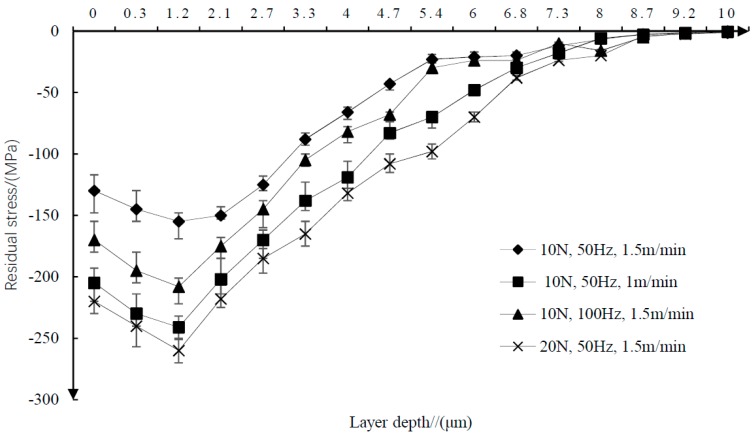
Layer depth of residual stress.

The thickness of the residual stress layer measured by the X-ray projector is shown in Figure 4. When the surface depth is less than 1.2 μm, the residual compressive stress increases with the increase of the surface depth, and decreases with the decrease of the surface depth.

**Figure 5:**
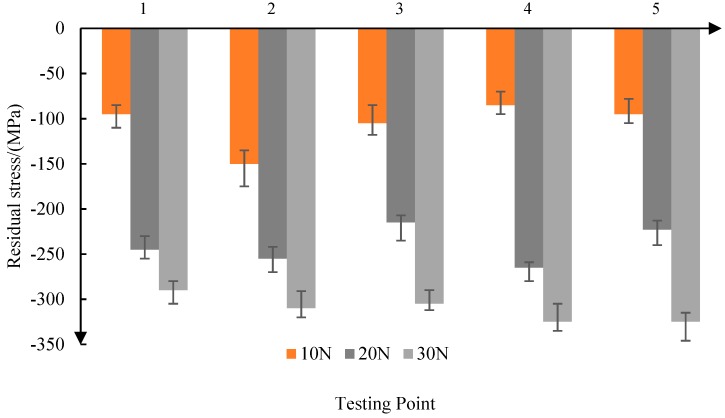
Influence of contact force on residual stress.

As shown in Figure 5, when the contact force is 10 N, the average residual stress is −106 MPa. When the contact force is increased to 30 N, the average residual stress is −311 MPa. Therefore, the residual compressive stress increases with the increase of the contact force. At the same time, with the increase of the contact force, the consistency of the residual compressive stress is improved.

**Figure 6:**
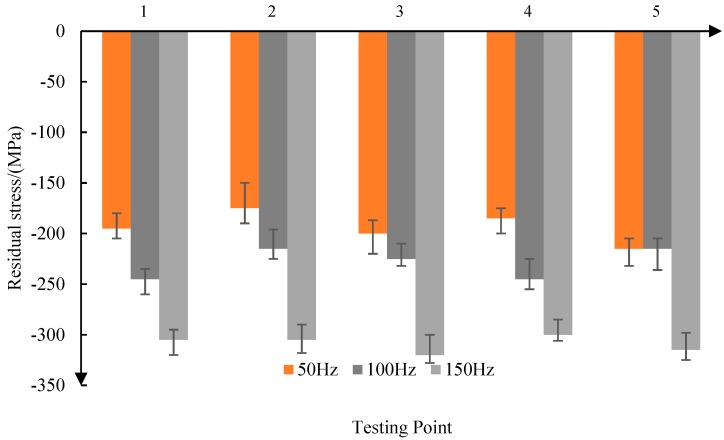
Effect of reciprocating frequency on residual stress.

As shown in Figure 6, when the reciprocating frequency is 50 Hz, the average residual stress is −194 MPa, and when the reciprocating frequency increases to 150 Hz, the average residual stress is −309 MPa. So, the residual compressive stress increases with the increase of the reciprocating frequency. At the same time, with the increase of the reciprocating frequency, the consistency of the residual compressive stress is improved.

**Figure 7:**
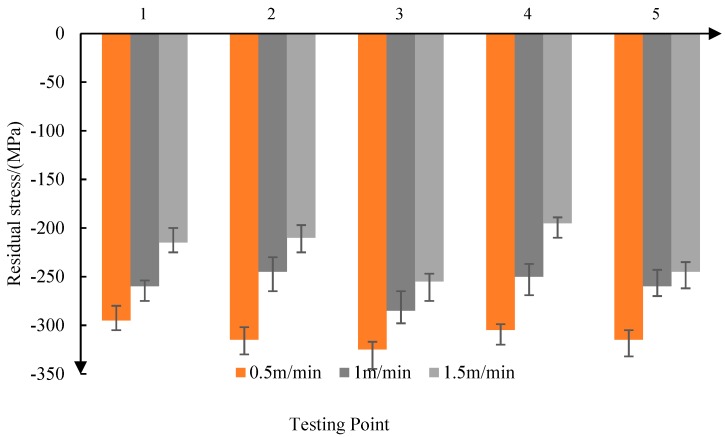
Effect of feed rate on residual stress.

As shown in Figure 7, when the feed rate is 0.5 m/min, the average residual stress is −311 MPa. When the feed velocity increases to 1.5 m/min, the average residual stress is −224 MPa. So, the residual compressive stress decreases with the increase of the feed rate. At the same time, with the decrease of the feed speed, the consistency of the residual compressive stress is improved.

From the above analysis, it can be seen that the residual compressive stress produced by abrasive belt grinding is in the range of 120–300 MPa. With the increase of grinding contact force, the increase of reciprocating frequency, and the decrease of feed speed, the residual compressive stress on the surface of the parts increases.

## 4. Analysis of Surface Residual Stress Characteristics

### 4.1. Finite Element Analysis

**Figure 8:**
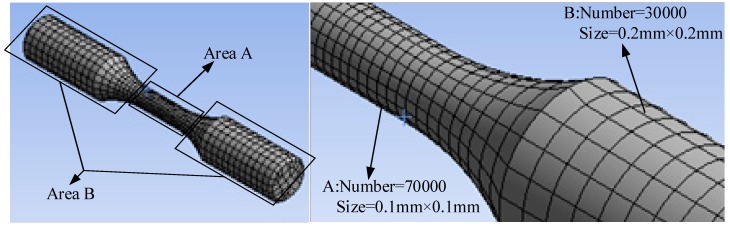
Element division.

Because this model is regular and simple in shape, the sweep method is used to divide it. The meshes are hexahedron, the middle meshes are fine, and the ends are wide, so that the meshes and the fatigue life of the middle sections can be calculated more quickly and accurately, as the middle is the position to analyse. The details of the element division are shown in Figure 8.

**Figure 9:**
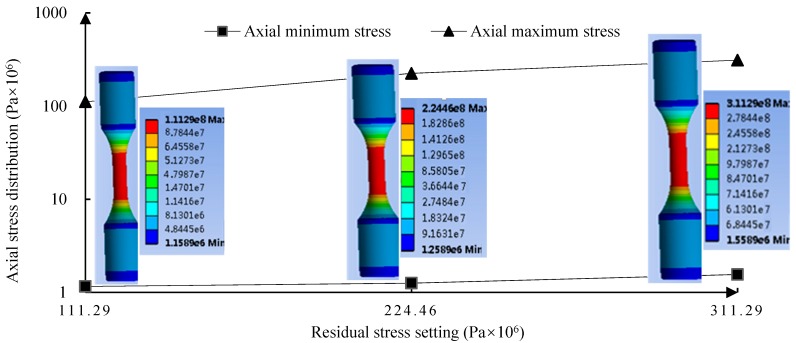
Stress distribution after normal stress is applied.

When the tensile force is applied to the middle surface of the bar, the stress value of the intermediate surface increases significantly under the pressure, which indicates that the tensile stress of the middle surface is superimposed with the load applied at both ends, thus increasing the stress value of the intermediate surface. This is because the applied residual stress can neutralize a part of the load acting on the intermediate surface to a certain extent. By applying the normal tensile stress, the simulation residual stress values of the surface are found to be 110 MPa, 220 MPa, and 310 MPa, respectively, as shown in Figure 9.

### 4.2. Analysis of Fatigue Life

**Figure 10:**
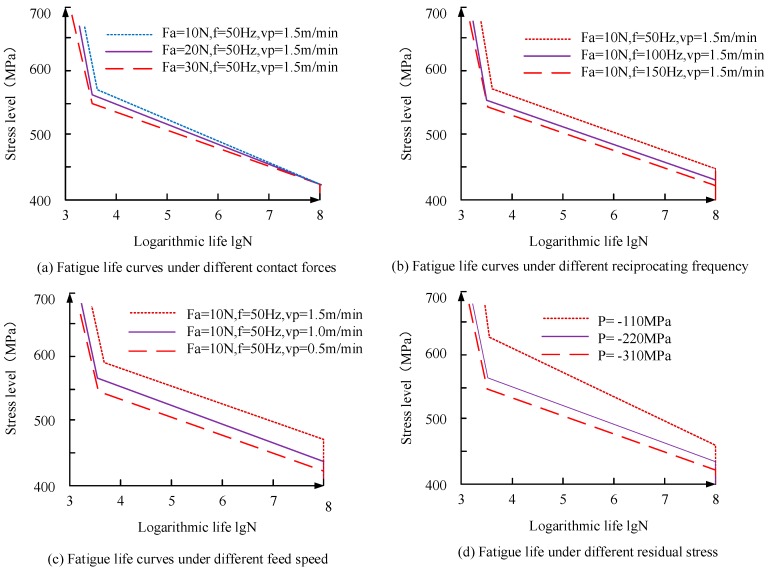
Fatigue life curves: (**a**) under different forces on the surface; (**b**) under a different reciprocating frequency; (**c**) under different feed speed; (**d**) under different residual stress.

(1) The ANSYS Workbench software material library provides the common fatigue data of iron, magnesium alloys, titanium alloys, and so on, mainly including the general data information of the material, the fatigue life curve of high and low cycles, and so on. The properties of the simulation materials are set according to Table 1 and Table 2. The S–N curves (the fatigue strength of the material standard specimen is the ordinate, and the logarithmic value lg N of the fatigue life is taken as the transverse coordinate; the curve of the relationship between the fatigue strength and the fatigue life of the standard specimen under certain cyclic characteristics is expressed) of TC17 are set according to the experimental data.

(2) The loading mode used in this experiment is the stress control, the cyclic stress spectrum of the sinusoidal pulse is used, and the cyclic characteristic stress ratio, *R*, is 0.1, and the frequency is 50 Hz. By changing the scale factor to enlarge and reduce the force of the static analysis in front, we can obtain the desired stress values, and thus carry out the fatigue simulation experiment under different stress levels.

(3) Figure 10 shows the fatigue life curves of titanium alloy bars under different residual stresses under varying grinding process parameters. The process parameters are as follows: the contact force is 10 N, 20 N, and 30 N; the reciprocating frequency is 50 Hz, 100 Hz, and 150 Hz; and the feed speed is 0.5 m/min, 1.0 m/min, and 1.5 m/min, respectively.

As can be seen from Figure 10a, with the increase of the grinding contact force, *F*_a_, the fatigue life of 550 MPa increases from 9.97 × 10^6^ cycles to 5.5 × 10^7^ cycles when the reciprocating frequency, *f*, is 50 Hz and the feed speed *v*_p_ is 1.5 m/min. Figure 10b shows that when the contact force, *F*_a_, is 10 N and the feed velocity, *v*_p_, is 1.5 m/min, the fatigue life increases from 9.97 × 10^6^ cycles to 3.32 × 10^7^ cycles. With the increase of the grinding reciprocating frequency, the fatigue life increases from 9.97 × 10^6^ cycles to 3.32 × 10^7^ cycles under the tensile loading level of 550 MPa, and it can be seen from Figure 10c that when the contact force, *F*_a_, is 10 N and the reciprocating frequency, *f*, is 50 Hz, the fatigue life decreases with the decrease of the feed speed. The fatigue life increased from 9.97 × 10^6^ cycles to 3.98 × 10^7^ cycles under the tensile load level of 550 MPa. It can be seen from Figure 10d that the greater the residual compressive stress, the greater the fatigue life at the same load stress level. The curve is vertical at 1 × 10^8^ cycles, which is attributable the maximum fatigue life of 1 × 10^8^ cycles when the S–N curve of TC17 is set in the material library, so the software takes 1 × 10^8^ cycles as the limit fatigue value in the analysis. 

It can also be seen from Figure 10 that when the working stress is about 550 MPa, the slope of the fatigue life curve changes, and with the increase of residual compressive stress, the point of slope change of the fatigue curve moves upward, relative to the axis. The reason for this is that when the work stress increases, the fatigue crack initiation on the surface has a more obvious stress concentration; so, it has less fatigue strength and tends to have a bigger slope, which causes less cycles. When the surface compressive residual stress increases, the crack initiation appears from the surface to the subsurface, where it tends to have less ability to take the place of the stress concentration; thus, at the same stress level, it can bear more load cycles.

In general, the residual stress is mainly divided into two types, residual compressive stress and residual tensile stress. In general, the so-called residual stress is called residual tensile stress, which exists as an outward pressure on the surface of the workpiece. This is an undesirable force that plays a significant role in the deformation of the workpiece, the initiation of cracks, the cracking, and surface wear; and the residual compressive stress is a desirable residual stress, which is the opposite to the tensile stress. It exists on the surface of the workpiece as an inward pressure, rather than as an outward tension. Therefore, if there is residual compressive stress on the surface of the workpiece, the fatigue strength and fatigue life of the workpiece will be greatly improved, and the wear resistance and corrosion resistance will be much better [31].

### 4.3. Fatigue Test Analysis 

#### 4.3.1. Fatigue Test

(1) Fatigue Test Equipment and Parameters

The experiment was carried out on a MTS809-10T tension torsion static fatigue test system (MTS809-10T, MTS, Eden Prairie, MN, USA). The system is mainly composed of an 809 hydraulic servo material test bench, a tension and torsion compound load sensor, a side load static actuator, and a MTS TESTSTAR digital control system. The main technical indicators are as follows: maximum dynamic pull, 100 kN, and torque, 1000 NM. The size of the standard specimen is as follows: the diameter of the round rod specimen is 6–30 mm, and the thickness of the plate specimen is less than 12 mm and the width is less than 130 mm. The load we used in this experiment was sinusoidal, with a frequency of 50 Hz and a cyclic characteristic stress ratio, *R*, of 0.1. The value of the loads were 450 MPa, 475 MPa, 500 MPa, and 525 MPa, respectively.

(2) Fatigue Test Data

The fatigue data obtained from the tensile tests on the specimens with different surface residual compressive stress (measured by X-ray) using the testing machine are shown in Table 3. From the eight groups of data, we can see that the measured experimental data are in line with the reality that a specimen that does not necessarily need to be removed because of defects in the material and processing process. Under the same working stress, the fatigue life increases with the increase of residual compressive stress, and the fatigue life decreases with the increase of working stress, under the same residual compressive stress.

#### 4.3.2. Simulation Experiment Data Analysis

**Figure 11:**
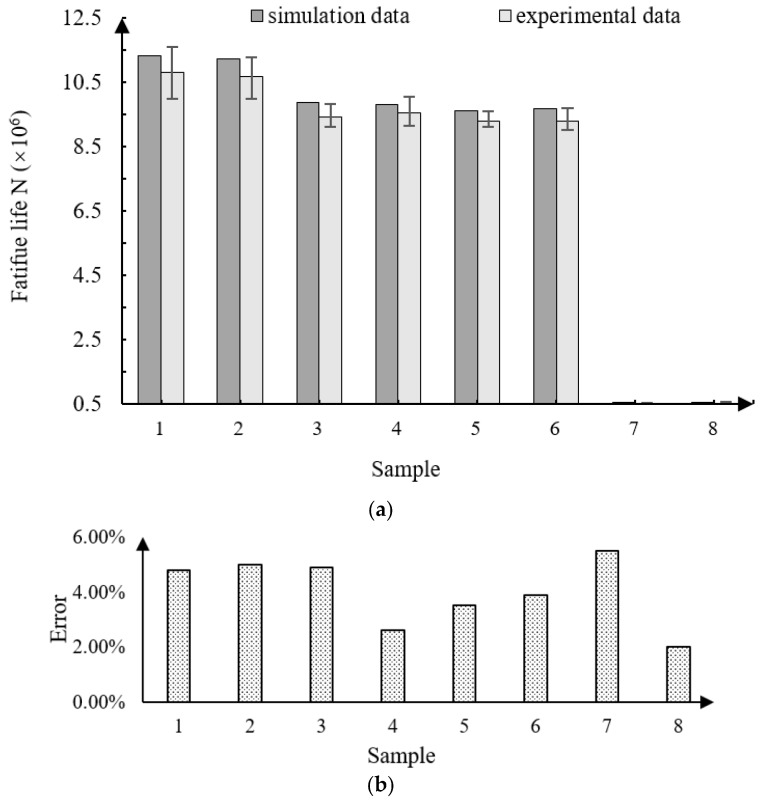
Simulation data and experimental data error. (**a**) Comparison of simulation data and experimental data; (**b**) error between simulation data and experimental data.

The data of the fatigue life from the simulation and experiment were compared and analyzed. As a whole, the error between the experimental data and our simulation results, as shown in Figure 11b, is less than 10%, which further illustrates the appropriateness of the simulation.

It can be seen from Figure 11a that the fatigue life according to the simulation data is always higher than the actual experimental data, which is because, in addition to the effects of roughness, surface hardness, residual stress, and other stress levels, there are also various uncertainties in the experimental environment, such as temperature changes, the imprecise measurement of integrity parameters, and so on. The lifetimes of samples 7 and 8 were significantly lower than that of the other samples, because the working stress of these two samples’ groups was the highest, and the compressive residual stress on the surface was the lowest. According to the previous simulation experiments, we can see that the increase of the surface compressive residual stress can increase the fatigue life, and the increase of the working stress at both ends can reduce the fatigue life. The comparison between samples 6 and 7 and samples 5 and 6 shows that the increase of working stress is 25 MPa, while the increase of residual stress is 80 MPa on average, but the fatigue life between them has little difference. The fatigue life of samples 5 and 6 is about 2% higher than that of samples 6 and 7. It can be seen that in this experiment, the influence of working stress on fatigue life is greater than that of residual compressive stress.

#### 4.3.3. Analysis of Fatigue Fractures

**Figure 12:**
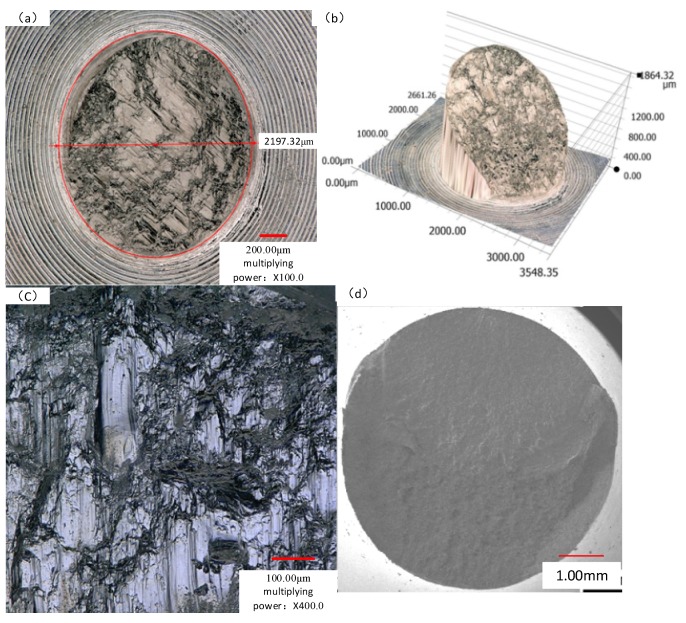
Final fracture area macro map: (**a**) radial microscopic observation; (**b**) microscopic size distribution around a plastic fracture; (**c**) macroscopic map of the final fault zone; (**d**) macroscopic fracture surface.

The macroscopic morphology of a fatigue fracture is divided into the following three parts: the fatigue source (source), crack propagation (propagation), and final fracture (transient fracture). A typical tested bar is chosen for the analysis, and the residual stress is 300 MPa under the working stress of 500 MPa. Figure 12 shows the microscopic figures of the final fracture zone. It can be clearly seen from Figure 12b that the strength limit of the material is exceeded in the transient fault area, because the area under force is too small to cope. The plastic deformation fracture occurs in the middle part, and the shear port presents a 45° angle and slip separation. In Figure 12c, the material is slip-fractured during the deformation in Figure 12b. The macroscopic fracture surface is smooth in Figure 12d. This is due to repeated extrusion friction at crack initiation under high cycle fatigue.

**Figure 13:**
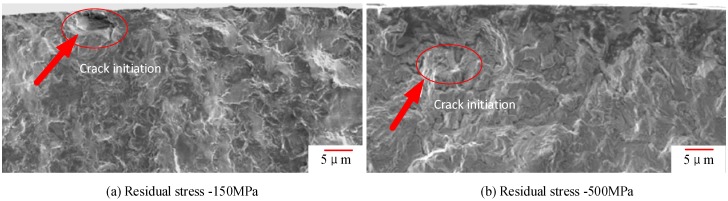
Fatigue crack initiation under different residual stresses in a cross-section view: (**a**) under the residual stress of −550 MPa; (**b**) under the residual stress of −500 MPa.

**Figure 14:**
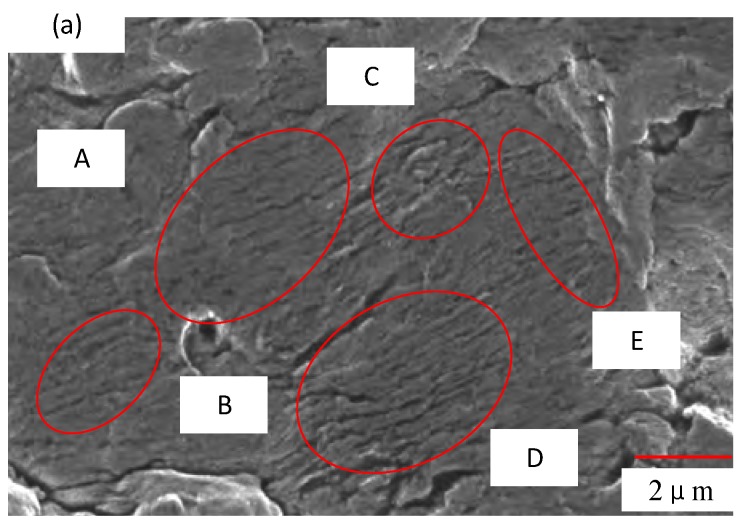
Fatigue crack growth.

Figure 13 shows that under the same stress level and different residual compressive stress, the fatigue crack initiation point is different; the arrows indicate where the fatigue crack initiation occurs at the residual compressive stress level. It can be seen from Figure 13a that the fatigue crack initiation point is on the surface of the specimen under low residual compressive stress, and that the fatigue crack initiation occurs on the subsurface of the specimen under high residual pressure, as shown in Figure 13b. The results show that the larger the residual compressive stress is, the less the fatigue crack initiation occurs on the surface, and the better the surface quality is. Figure 14 shows the TC17 fatigue crack growth zone, from which we can see the typical fatigue bands (areas A, B, C, D, and E). Obvious secondary crack characteristics have been found.

**Figure 15:**
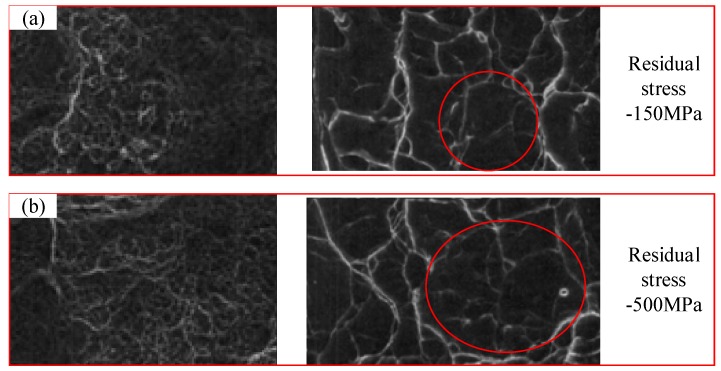
SEM fracture morphology: (**a**) under the residual stress of −150 MPa; (**b**) under the residual stress of −500 MPa.

The port morphology of the fatigue specimen was observed using SEM, as shown in Figure 15. The center of the port exhibited a ductile fracture, which was characterized by typical uniaxial tensile equiaxed dimples. There were no obvious nucleation particles in the dimples, and they were all small equiaxed dimples. There are a few large dimples, and these dimples contain a few smaller dimples, and some dimples have tiny holes in them. The fracture morphology at room temperature is of a ductile fracture and fine equiaxed dimples. The dimples in Figure 15b are larger than those in Figure 15a. The bigger dimple size confers a good plasticity. The larger the residual stress is, the larger the dimple is and the better the surface is. So, the increase of residual stress can improve the plasticity to some extent.

## 5. Conclusions

In this paper, the residual stress of a TC17 titanium alloy after belt grinding and its impact on the fatigue life were studied.

Firstly, the residual stress produced by grinding the titanium alloy surface was found to be between −120 and −300 MPa. The residual compressive stress increases with the increase of depth when the surface depth is less than 1.2 μm, and when the surface depth layer is greater than 1.2 μm, the residual compressive stress decreases with the increase of the surface depth. The experimental results show that with the increase of the grinding contact force, the increase of the reciprocating frequency, and the decrease of the feed speed, the residual compressive stress on the surface of the parts increases, and the fatigue life is higher at the same working stress level. Based on Bragg’s law, the surface residual stress is represented by normal tension and pressure on the surface of the bar in order to simulate the residual stress, and then the residual stress model is simulated using the tensile force on the surface of the model.

Then, the fatigue life of the bar subjected to a sinusoidal tensile load at both ends was analyzed by simulating the fatigue test of the titanium alloy bar. The fatigue life of the same working stress level increased with the increase of the grinding contact force. With the increase of the reciprocating frequency, the fatigue life of the same working stress level increased, and with the decrease of the feed speed, the fatigue life of the same working stress level was higher. 

The slope of the fatigue life curve changes at about 550 MPa, and with the increase of residual compressive stress, the point of slope change of the fatigue curve moves upward relative to the axis. The reason for this is that when the working stress increases, the fatigue crack initiation on the surface has a more obvious stress concentration, and thus has a lower fatigue strength and tends to have a bigger slope, which means fewer cycles to fatigue. When the surface compressive residual increases, the location where the crack initiation begins shifts from the surface to the subsurface, where it tends to have less ability to take the place of the stress concentration; thus, at the same stress level, it can bear more load cycles.

Finally, fatigue experiments were carried out and the fracture surface was analyzed. The error between the simulation data and the experimental data was less than 10%, and the fracture morphology at room temperature was of a ductile fracture and fine equiaxed dimples. The greater the residual compressive stress is, the better the surface quality and the less apparent the fatigue crack initiation point is on the surface. From the propagation, we can see the typical fatigue bands and obvious secondary crack characteristics.

## Figures and Tables

**Table 1:** Material composition.

**Component**	Al	Sn	Zr	Mo	Gr	Ti
**Content (%)**	5%	2%	2%	4%	4%	balance

**Table 2:** Material physical properties.

Elastic Modulus (GPa)	Elongation (%)	Shrinkage (%)	Density (kg/m^3^)	Yield Strength (MPa)	Tensile Strength (MPa)
111.5	10	17.5	4640	1110	1180

**Table 3:** Fatigue life of specimens with different surface integrity and stress levels.

Sample	Residual Stress (MPa)	Working Stress (MPa)	Fatigue Life (Cycle)	Logarithmic Life (lg N)
1	−284	450	11,324,681	7.05402
2	−250	450	11,223,465	7.05012
3	−214	475	9,876,522	6.99460
4	−202	475	9,798,325	6.99115
5	−287	500	9,628,951	6.98357
6	−298	500	9,668,362	6.98535
7	−147	525	542,957	5.80818
8	−150	525	547,132	5.73809
